# Bifocal rupture of the patellar tendon in TKA: Case report and review of the literature

**DOI:** 10.1016/j.ijscr.2024.109231

**Published:** 2024-01-10

**Authors:** F. Lamnaouar, A. Rajaallah, N. Nkeshimana, A. Lahjouji, M. Rahmi, M. Rafai

**Affiliations:** a Resident in Orthopedics and Traumatology Surgery, 32 Pavilion of CHU Ibn Rochd, Casablanca, Morocco; b Professor in Orthopedics and Traumatology Surgery, 32 pavilion of CHU Ibn Rochd, Casablanca, Morocco

**Keywords:** Patellar tendon, Quadriceps tendon, Total knee arthroplasty, Bifocal rupture, Tendon augmentation, Tendon repair

## Abstract

**Introduction and importance:**

Rupture of the extensor apparatus is a serious complication that can occur in a prosthetic knee. Bifocal extensor ruptures are rare and even more uncommon in adults.

**Case presentation:**

We report the case of an obese, diabetic, and hypertensive patient who underwent total knee arthroplasty two weeks previously and was admitted following a fall from her height for a rupture of the patellar tendon with release of the sutures. An investigation revealed bifocal avulsion of the patellar tendon from its patellar and tibial insertion. The patient was treated with double lacing and anchoring at the patellar and tibial levels with wire cerclage.

**Clinical discussion:**

Several factors contribute to tendon fragility: degenerative changes linked to age, general or local conditions, and surgical approaches for TKA. These abnormalities also affect the tendon's ability to heal and should suggest the inadequacy of simple repair without tendon augmentation using a plasty, an autograft, or an allograft.

**Conclusion:**

The prognosis for patients with extensor tendon ruptures in the TKA (total knee arthroplasty) remains unclear, and in our case, this was exacerbated by the delay between prosthesis insertion and the incident and by the open nature of the lesion.

## Highlights

• Weakening of the extensor apparatus in the suites of the total knee arthroplasty is a dramatic complication.
• Surgical exposure is incriminated in the interruption of the vascularization of the extensor apparatus, making it fragile and predisposing it to injuries such as patella fractures and ligament ruptures, and also affecting its ability to heal following an action of repair or reconstruction.
• The damage of the vascularization of the extensor apparatus affect also its capacity to heal which explains the necessity of a plasty or a reinforcement
• The bifocal rupture of the extensor apparatus is a rare entity, even more in the old population.

## 1 Introduction

Patellar tendon rupture is a serious complication that can occur after TKA(total knee arthroplasty) and has a frequency of 1 %. It may be traumatic or nontraumatic in nature. In both cases, the factors causing tendon fragility can be vascular, essentially related to the surgical approach; mechanical due to possible conflicts with prosthetics; other factors related to the terrain; and certain conducive anomalies of the native knee [1].

Ruptures of the extensor mechanism are common injuries, but bifocal ruptures are rare. They are more common in children. The clinical features of this type of injury vary according to age in terms of circumstances, mechanism, and appearance, with proximal and distal patellar tendon rupture being extremely rare in adults [2].

Surgical options include direct repair, reconstruction with allograft using the Achilles tendon, total extensor system “tendon-bone–tendon graft”, or a plasty using the semitendinosus muscle [3].

We report a rare case of bifocal rupture of the patellar tendon, with a never-described variety, the rupture was accompanied by loosening sutures making it an open rupture, after a review of the literature our management could have been better than what was done.

## 2 Observation

The reporting of this work follows the SCARE checklist criteria [13], ensuring adherence to guidelines for quality reporting in case series.

**Fig. 1:**
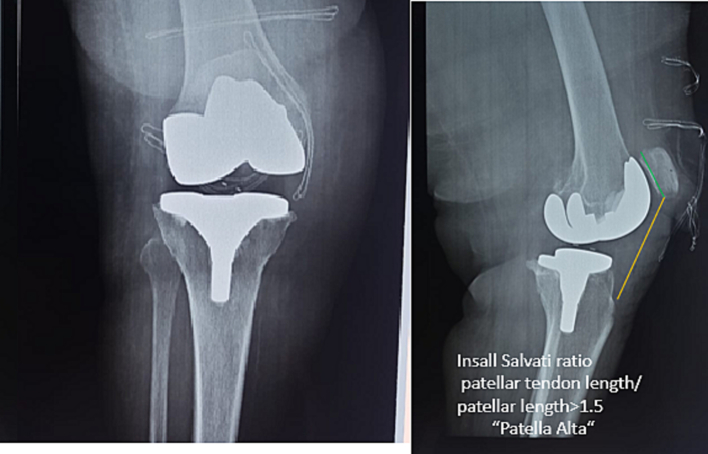
X-ray of the knee on admission: yawning on the medial side, deviation radiopaque markers of the polyethylene, and elevation of the patella in profile the Insall Salvati ratio is equal to 1.6 synonym of patella alta.

We report the observation of a 77-year-old patient with a BMI of 31 kg/m^2^ and type 2 diabetes and hypertension who was treated 11 days previously for tri-compartmental gonarthrosis of the right knee with a posterior-stabilized total knee prosthesis without resurfacing of the patella. Two hours before admission, the patient fell from a height due to carelessness, landing directly on the prosthetic knee. On admission, there was a loose suture with a tendon stump, with no downstream vasculo-nervous disorder. Radiological examination revealed a yawning joint, deviation of the radio-opaque polyethylene markers, which led to suspicion of injury to the medial collateral ligament, dislocation of the mobile plate, and the appearance of a high patella, the Insall salvati ratio calculated was superior to 1.5 (Fig. 1).

**Fig. 2:**
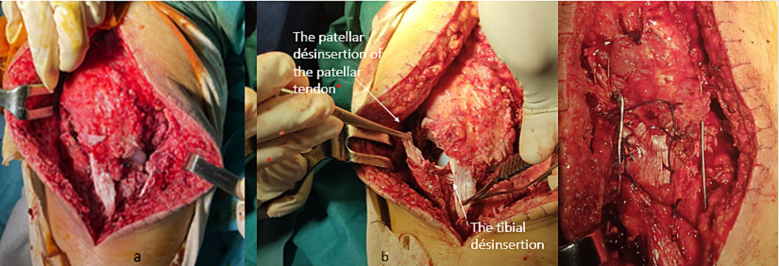
a, b per operatoir aspect of the Bifocal rupture of the patellar tendon. c. Surgical repair with suture and trans-bone anchoring.

**Fig. 3:**
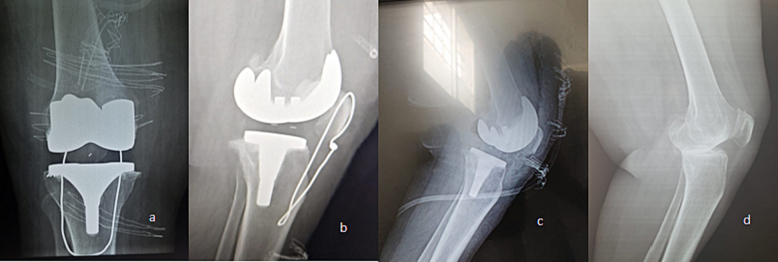
a, b. X-ray views after repair. c. x-rays view after TKA before trauma. d. Lateral radiographic view of the contralateral knee to compare patellar height.

The patient was admitted urgently to the operating room, where exploration revealed no dislocation of the polyethylene, no fractured lesion, and no laxity in the frontal plane; she also had the wound decontaminated. There was also a bifocal avulsion of the patellar tendon separated by a longitudinal break: the lesion combined a patellar disinsertion of the external part of the tendon and a disinsertion of its medial part of the anterior tibial tuberosity with a bald appearance of the tuberosity (Fig. 2). The patellar tendon was repaired in three stages: externally, by lacing and anchoring with trans-bone tunnels at the level of the patella using Vicryl 2 (Krackow whip stitch); internally, by lacing and anchoring with trans-bone tunnels at the level of the anterior tibial tuberosity; and finally, by X-stitches for longitudinal rupture. The suture was protected by the application of a cerclage wire (Fig. 3).

The evolution of the surgical procedure was marked by the occurrence of an infection in the surgical wound, and the patient underwent reoperation with abundant irrigation of the wound and polyethylene replacement, which resulted in better clinical outcomes.

## 3 Discussion

Rupture of the patellar tendon is a serious complication that can occur following total knee replacement. The tendon may be subject to avulsion at the summit of the patella or to avulsion of the anterior tibial tuberosity, or rupture may occur in the entire tendon [4].

Acute rupture of the patellar tendon is uncommon, as it requires a high impact force, while chronic ruptures due to degenerative changes are frequent. [4]

With an incidence of 1–12 %, extensor rupture in total knee prostheses is a complication that radically changes the prognosis of the prosthesis. [4,5]

Being able to withstand tension forces that are greater than those applied to the tendon, the forces required to lead to a rupture should be greater than or equal to 17.5 times the body weight. The tensile forces exerted are less in the mid-substance of the tendon than in the patellar or tibial insertion zone [6].

**Fig. 4:**
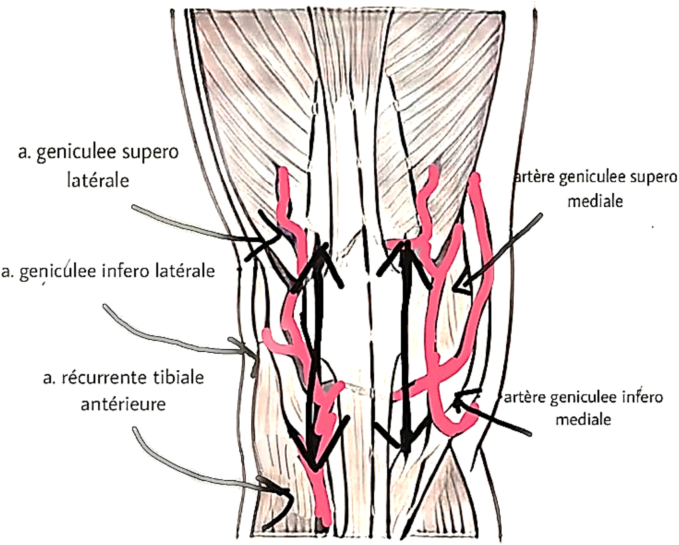
Diagram showing the vascularization of the extensor apparatus [1].

Surgical exposure is associated with factors that interrupt the vascularization of the extensor apparatus, making it fragile and predisposing it to injuries such as patellar fractures and ligament ruptures, affecting its ability to heal following repair or reconstruction. [1,5] Exposure via a medial parapatellar approach may damage the inféro-medial geniculate artery, while the release of the lateral retinaculum damages the superolateral geniculate artery [1,7]. The excision of Hoffa's fat risks further damage to the blood supply by affecting the anterior recurrent tibial artery (Fig. 4). [7]

Based on a scintigraphic study, the relationship between external release associated with the medial parapatellar approach was judged to be statistically significant (*p* = 0.031) by Pawar et al. [8]. The association between external release and osteonecrosis has been suggested by several authors, Cameron and Fedorkow [8] and Clayton and Thirpathi [8]. According to Pawar et al., this hypervascularization was transient because it was reversible after 8 weeks, which eliminated the possibility of long-term complications once this interval had elapsed. [8] A study published in 2019 by Quack et al. aimed to assess postoperative changes in the patellar and quadricipital tendons following TKR surgery. The study used ultrasonic shear wave elastography and other sonographic techniques (Doppler and B-mode). The conclusions were as follows: more neovascularization phenomena exist in operated tendons, and there is an alteration in tendon rigidity. Abnormalities can be detected up to 2 years postoperatively [9].

Other risk factors for patellar tendon rupture on TKA include a stiff and tight knee, abnormalities in patellar height, tibial or patellar prosthetic overhang, ascent of the joint space (IA) leading to impingement between the patellar tendon and the tibial plateau, and comorbidities such as diabetes, inflammatory rheumatism, and renal insufficiency [1,10].

**Fig. 5:**
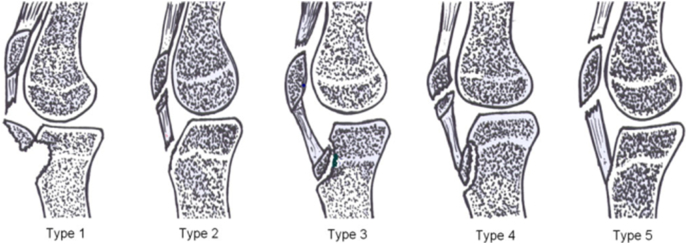
Classification of Kang et al. into 5 types [2]*.*

In a review of the literature on bifocal extensor tears, Kang et al. established a classification of five types [2]: type 1 consists of an avulsion of the tibial tuberosity with avulsion of the patellar tendon on the TTA; this is the most frequent form of tears, which occurs in young subjects between the ages of 12 and 16 years during physical activity such as jumping. Type 2 is an avulsion of the lower pole of the patella with avulsion of the patellar tendon on the TTA. Type 3 is an avulsion of the TTA with rupture of the quadricipital tendon. Type 4 is an avulsion fracture of the TTA with avulsion of the inferior pole of the patella. Type 5 is a rupture of the quadricipital tendon with avulsion of the patellar tendon from the lower pole of the patella (Fig. 5).

**Fig. 6:**
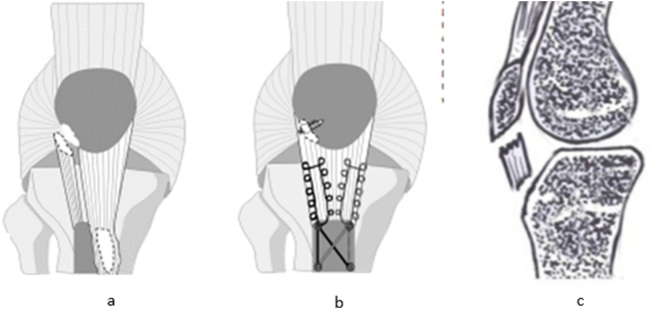
Schematic presentation of bifocal avulsion in the article of Hermansen et al. b the surgical repair by Hermansen [13] Schematic representation of the break in our observation.

Our observations can thus be classified in a sub-group of type 2 or type 6 on their own (Fig. 6).

This complication can be avoided during the surgical approach by taking special care during exposure, good soft tissue management and monitoring of the extensor apparatus [6].

Incomplete ruptures with an intact extensor apparatus should be treated by immobilization in full extension for 2 weeks, followed by progressive recovery of joint amplitudes with active flexion and passive extension for 6 weeks, with strengthening commencing after the sixth week. Surgical treatment is indicated in the event of a deficit in the extensor apparatus, whatever the type of damage, age or degree of activity. Immediate repair is always preferred [9].

For surgical treatment, there are several options: simple repair, repair with reinforcement using a neighboring tendon, reconstruction using an artificial ligament, allograft replacement or salvage techniques such as the use of the gastrocnemius flap and knee arthrodesis [1].

Patellar tendon wounds are treated as open trauma, with antitetanus prophylaxis, trimming and restoration of tendon continuity. Some authors even recommend two-stage repair to avoid disunions due to sepsis. Repair will be carried out using a terminal suture reinforced by a peritendinous suture, closing the tendon sheath, which determines the long-term results. The suture will be protected by wire cerclage or even the use of tendon reinforcement via an autograft [11].

However, the prognosis for this type of lesion is always a problem. In a Mayo Clinic study of 367 TKAs that were revised for extensor problems, 23 % required reoperation [12].

## 4 Conclusion

Rupture of the patellar tendon is a serious complication when it occurs in a prosthetic knee, and the failure rate is not negligible in the event of revision. A total knee prosthesis leads to a weakening of the extensor apparatus due to the interruption of a large part of the blood supply. The surgeon will be obliged to re-establish the height of the joint space and avoid overhanging the parts to avoid the resulting complications. The authors agree on the need for tendon reinforcement each time tendon repair is carried out following a TKA.

## Consent

Written informed consent was obtained from the patient for publication of this case report. Accompanying images.

## Ethical approval

The study is exempt from ethnical approval in our institution for being just a case report only the patient consent is necessary (comité d’ethique du centre hospitalier IBN Rochd de Casablanca).

## Funding

No funding received.

## Author contribution

Dr Foad Lamnaouar: the operator, writing the paper

Dr Rajaallah Abdssamad reviewing the paper

Nkeshimana Nicodeme 2^nd^ operator / writing the paper

Dr Lahjouji Achraf writing the paper

Dr Rahmi Mohamed reviewing the paper

Dr Rafai Mohamed : reviewing the paper/ concept

## Guarantor

Dr Foad Lamnaouar

## Conflict of interest statement

No conflict of interest.
